# The effects of semantic congruency: a research of audiovisual P300-speller

**DOI:** 10.1186/s12938-017-0381-4

**Published:** 2017-07-25

**Authors:** Yong Cao, Xingwei An, Yufeng Ke, Jin Jiang, Hanjun Yang, Yuqian Chen, Xuejun Jiao, Hongzhi Qi, Dong Ming

**Affiliations:** 1 National Key Laboratory of Human Factors Engineering, China Astronaut Research and Training Center, Beijing, China; 2 Department of Biomedical Engineering, Tianjin University, Tianjin, China

**Keywords:** BCI, Speller, ERP, Audiovisual, Semantic congruency

## Abstract

**Background:**

Over the past few decades, there have been many studies of aspects of brain–computer interface (BCI). Of particular interests are event-related potential (ERP)-based BCI spellers that aim at helping mental typewriting. Nowadays, audiovisual unimodal stimuli based BCI systems have attracted much attention from researchers, and most of the existing studies of audiovisual BCIs were based on semantic incongruent stimuli paradigm. However, no related studies had reported that whether there is difference of system performance or participant comfort between BCI based on semantic congruent paradigm and that based on semantic incongruent paradigm.

**Methods:**

The goal of this study was to investigate the effects of semantic congruency in system performance and participant comfort in audiovisual BCI. Two audiovisual paradigms (semantic congruent and incongruent) were adopted, and 11 healthy subjects participated in the experiment. High-density electrical mapping of ERPs and behavioral data were measured for the two stimuli paradigms.

**Results:**

The behavioral data indicated no significant difference between congruent and incongruent paradigms for offline classification accuracy. Nevertheless, eight of the 11 participants reported their priority to semantic congruent experiment, two reported no difference between the two conditions, and only one preferred the semantic incongruent paradigm. Besides, the result indicted that higher amplitude of ERP was found in incongruent stimuli based paradigm.

**Conclusions:**

In a word, semantic congruent paradigm had a better participant comfort, and maintained the same recognition rate as incongruent paradigm. Furthermore, our study suggested that the paradigm design of spellers must take both system performance and user experience into consideration rather than merely pursuing a larger ERP response.

## Background

The technology of brain–computer interfaces (BCIs) provides a novel communication method for people with severe neuro-muscular diseases, such as severe paralysis and amyotrophic lateral sclerosis (ALS) [1, 2]. Specifically, a BCI system can translate signals generated by brain activities into control signals to allow command of specific devices that could help patients communicate with the external environment without the participation of muscles [3]. Compared with several common BCI systems, for instance, steady-state visual evoked potential (SSVEP) based BCIs and sensorimotor rhythms (SMR) based BCIs, event-related potential (ERP) based BCIs are generally considered to be more stable and more efficient [4], characteristics that have resulted in development of ERP-based applications being a primary control signal for BCI systems.

Speller is a typical BCI applications that aims at helping patients communicate with others by mental typewriting. Pioneers in this field have developed many classical ERP-based speller paradigms. Most of the paradigms in early researches were visual-based unimodal stimuli. For instance, a P300 based paradigm was proposed by Farwell and Donchin [5] in 1988, considered as the first realization of a BCI speller and still used today. In this paradigm, a 6*6 character matrix is presented on the screen, with a random sequence of 12 flashes consisting of six rows and six columns that constitutes an oddball paradigm [6], to evoke a P300 response. Besides, with the progress of ERP-based spellers, some unimodal paradigms based on other sensory stimuli, such as audio-only stimuli [7, 8], and tactile sense-only stimuli [9, 10] were proposed as well. Nowadays, hybrid BCI system slowly entered the vision of researchers [11, 12], especially multimodal stimuli paradigm combining auditory-and visual-based speller have attracted much attention from researchers [13–17]. For one thing, some researches pointed out multimodal stimuli based spellers can address the limitation of eye gaze ability [13, 18–20]. For another, some researchers found the system performance of audiovisual spellers were better than that of unimodal stimuli based spellers [14, 16]. To be more specific, Belitski et al. [16]. implemented an online experiment of multimodal stimuli combining audio and visual data, and compared the performance among an audiovisual stimuli paradigm, an audio-only stimuli paradigm, and a traditional matrix paradigm. The results indicated that the audiovisual stimuli-based speller performed better than the traditional matrix speller and the unimodal auditory stimuli-based speller. The same result was also found in Wang’s research [14]. Additionally, Wang indicated that the ERP of multimodal stimuli was significant stronger than that of each unimodal stimuli, and it was significantly different from the sum of ERPs of the two unimodal stimuli. An et al. [13]. explored audiovisual stimuli for gaze-independent BCI from the perspectives of both behavioral and ERP data. The result showed that there were significant difference in both performance and brain activity between multimodal and unimodal stimuli-based spellers.

Most of the existing studies of audiovisual spellers are based on semantic incongruent stimuli [13–16]. This is because incongruent stimuli requires more attention of participants from the perspective of neurophysiology, which may allow larger amplitude ERPs compared with congruent stimuli. A similar viewpoint was verified in Andres’ research [21]. However, there were still some studies focusing on semantic congruent spellers. For instance, Laurienti et al. [22]. indicated that semantic congruent stimuli can significantly reduce reaction time through an experiment that used two circles colored red or blue as visual stimuli on combination with the pronunciation of ‘red’ and ‘blue’ as audio stimuli. A speller based on semantic congruent stimuli involving visual and spoken numbers was proposed [14], and its system performance was as good as that of traditional single modal stimuli based spellers. Overall, various approaches of multimodal based paradigm combining auditory and visual stimuli have been developed. However, there has not yet been a study that compares congruent stimuli and incongruent semantic stimuli from the perspective of the BCI speller.

With the exception of system performance, the user comfort of spellers is also becoming a higher priority for researchers. Earlier studies demonstrated the potential for physical and mental discomfort [23] for long-time use of spellers. Besides, semantic congruency may cause some psychological effects on the comfort, fatigue and mental workload of users. Thus, from the perspective of ergonomics, a well-designed speller should give consideration to both system performance and user comfort. This study focused on this issue, and attempted to compare the congruent and incongruent paradigms from both the aspect of behavioral data and ERP, to gain insight into the effect of semantic congruency on audiovisual stimuli paradigm based spellers.

## Method

### Participants

11 healthy participants (5 females) aged 22–33 (mean ± SD, 23.9 ± 1.14 years) took part in the experiment. All of the participants had normal hearing and normal or corrected-to-normal vision. All participants provided written informed consent prior to the experiment, and they volunteered to take part in the experiment.

### Stimuli

**Fig. 1:**
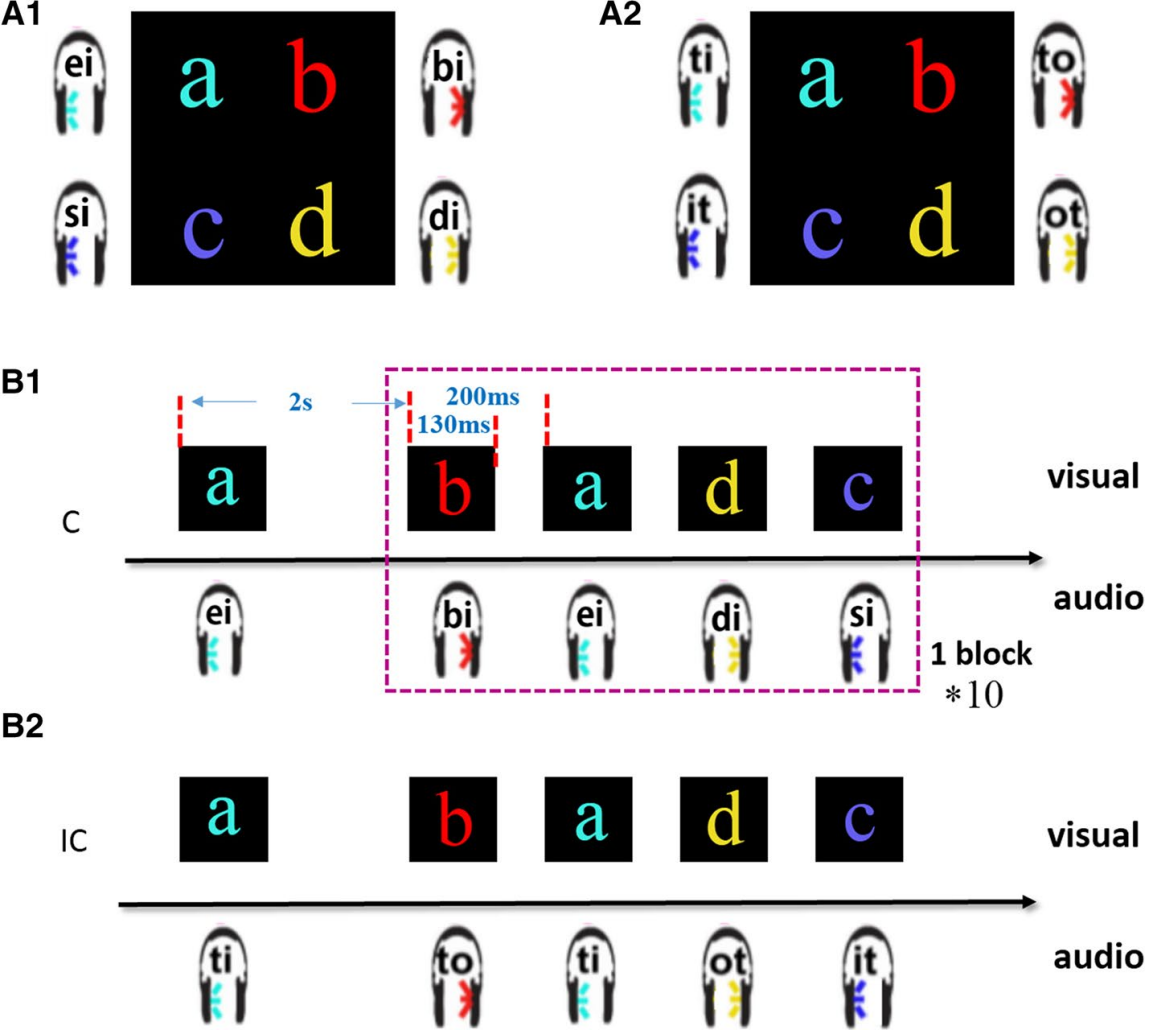
Stimuli and experimental procedure. ‘C’ and ‘IC’ are the abbreviation for congruent and incongruent, respectively. **A1**, **A2** show the stimuli of the two experiment paradigms, and **B1**, **B2** demonstrate the experimental procedure of the two paradigms. **A1**, **B1** represent the stimuli and procedure of semantic congruent paradigm, **A2**, **B2** represent the stimuli and procedure of the semantic incongruent paradigm. The coordinate axis in (**B1**), (**B2**) represent time. The first character means the target character in the following block and 2 s means the time interval between the target prompting and the block onset in (**B1**), (**B2**). Note that the four different random stimuli sequence consisted of blocks repeated 10 times. The visual stimuli and the auditory stimuli were presented simultaneously

Two paradigms combining visual and audio were adopted in this experiment. The visual stimuli were the same between the two audiovisual stimuli paradigms, however, the audio stimuli had two different choices. For the semantic congruent paradigm, the pronunciation of each audio stimuli was congruent with the corresponding visual stimuli. For instance, if the current visual stimuli was the letter “a”, the corresponding audio stimuli was the syllable ‘ei’ (the pronunciation of the letter ‘a’), as shown in Fig. 1(A1). For the semantic incongruent paradigm, the audio stimuli had little relationship with the visual stimuli letters, as shown in Fig. 1(A2). For both models, all audio stimuli and the corresponding visual stimuli were presented simultaneously.

Visual stimuli contained four different letters: ‘a’, ‘b’, ‘c’, ‘d’, and each letter had a unique color as Fig. 1 shows. These letters were presented in a random sequence at the center of a 19′ TFT screen with a refresh rate of 60 Hz. The inter stimulus interval (ISI) between two adjacent stimuli was 200 ms with a duration of 130 ms (the same as the duration of an audio stimuli).

Audio stimuli consisted of two conditions, For semantic congruent audio stimuli, we selected four short spoken syllables (‘ei’, ‘bi’, ‘si’, and ‘di’) to match the visual stimuli, with ‘ei’ and ‘si’ on the left channel, ‘bi’ and ‘di on the right channel. For semantic incongruent condition, the syllables ‘ti’, ‘to’, ‘it’, and ‘ot’ used in previous studies [24, 25] were adopted, with ‘ti’, ‘it’ on the left channel and ‘to’, ‘ot’ on the right channel. The duration of each stimulus was also 130 ms, and the ISI was 200 ms in both conditions. The audio stimuli were presented through comfortably positioned in-ear headphones, as Fig. 1 shows.

### Experimental procedure

The experimental paradigm was implemented in e-prime 2.0. Participants were instructed to sit in a comfortable position and keep their eyes staring at the center of the screen, with minimum eye movements or any other muscle artifacts throughout the whole experiment. Experiment for each participant consisted of two paradigms (semantic congruent paradigm and semantic incongruent paradigm). Each paradigm repeated twice, comprising four blocks. In each block, a random sequence of four single trial stimuli was repeated ten times. The experiment lasted about 8 min, and then the subjects were asked to relax for 5 min, before repeating the experiment.

At the end of the experiment, all participants were asked about which stimuli paradigm they preferred based on their participant comfort. In detail, evaluation of comfort in this study mainly took into account three questions: which paradigm is more difficult; which paradigm makes you feel more fatigue; which paradigm will you choose if there is another experiment. When a paradigm was chosen in two or three of the three questions, it was assumed the participant preferred this paradigm. There is currently no single evaluation method of participant comfort. Garcia et al. [26] compared the user comfort of different P300 speller configurations with NASA Task Load Index (NASA-TLX), while Ekandem et al. [27] evaluated the user comfort with the duration of BCI use. These studies considered only one factor of comfort evaluation, neither of them was the best choice. Our study considered the difficulty degree of experiment, the fatigue degree of participants and the willingness of re-participating in the experiment comprehensively in comfort evaluation, which was a more convincing choice.

### Data acquisition and preprocessing

The EEG signal was recorded from 64 Ag/AgCl scalp electrodes placed according to the positions of the extended International 10–20 system, and amplified by a Neuroscan NuAmp amplifier with a sampling rate of 500 Hz. During the data acquisition, all channels were referenced to the nose tip, with the ground electrodes placed at the frontal area neighboring the AFz channel. Electrode impedances were kept below 10 KΩ during data acquisition.

In data preprocessing, the raw EEG data were first re-referenced to the average signal of the left and right mastoid. Next, eye potential and motion artifacts were removed through the method of independent component analysis (ICA). After removal of eye potential and motion artifacts, the data was filtered with a 0.5–40 Hz band-pass filter, and down-sampled to 200 Hz for further analysis. Finally, the epoch data were extracted from −200 to 800 ms after each stimulus onset and the baseline was removed by subtracting the mean value between −200 and 0 ms.

### Classifiability and classification algorithm

For better classification performance, the channels and the features used for offline classification were selected based on classifiability analysis. Classifiability is a parameter indicating the difference between target and non-target stimuli, and it is usually expressed by the r^2^-value [28] defined as

1 $$r^{2} = \left( {\frac{{\sqrt {M_{T} M_{N} } \;\left( {\text{mean} \left( {X_{T} } \right) - \text{mean} \left( {X_{N} } \right)} \right)}}{{\left( {M_{T} + M_{N} } \right)\;\text{std} \;\left( {X_{T} \bigcup {X_{N} } } \right)}}} \right)^{2}$$

where, *M*_*T*_ and *M*_*N*_ are the sample size of the target and non-target respectively; *X*_*T*_ and *X*_*N*_ are the selected features vector of the target and non-target respectively.

Usually, the classification performance of speller depends not only on the amplitude of ERP data, but also on the classifiability of selected ERP features between target and non-target stimuli. Thus, the analysis of r^2^-value can provide the mathematic foundation for selecting channels and the features of each channel. Specifically, ERP data of 3 time intervals of 180–280, 300–450, and 480–530 ms down-sampled to 40 Hz with the greatest average r^2^ value of 10 channels (Cz, Pz, CPz, Oz, PO7, PO8, FC1, FC2, FC5, and FC6) based on the whole dataset were selected as features. Thus, 10*12 = 120 features for each stimuli were used for classifying.

Two classic classification algorithms, support vector machine (SVM) and stepwise linear discriminant analysis (SWLDA) were implemented for the binary classification. These two algorithms have been shown to be the most effective classifiers in previous speller study [29]. The SVM approach shows many unique advantages in solving small sample, nonlinear, and high dimensional pattern recognition problems. Two patterns are separated by SVM with a hyper-plane that has the maximum distance from the two patterns. Linear learning kernel was chosen for the SVM model since previous study indicated that linear kernel had a better performance compared with nonlinear method like Gaussian kernel [29], and the penalty coefficient was optimized by tenfold cross validation when training the SVM model. The algorithm has previously been implemented in LibSVM [30]. SWLDA is an extensive algorithm based on LDA. SWLDA can select the features used for calculation for better classification performance compared with LDA. To predict the target label, input features for analysis were weighted by ordinary least-squares regression. In this way, at last 60 features were selected for final analysis with the union of backward and forward stepwise calculations [29, 31]. Besides, three quarters of the total data was used for training the classifier, and the remaining one quarter was used for testing.

To investigate differences in performance between the congruent and incongruent paradigm in detail, single trial accuracy, character accuracy, and the recognition rate with increased repetition were compared by bootstrap *t* test.

### Statistical method

Bootstrapping-based *t* tests and ANOVAs were performed to analyze the effect of the semantic congruency on system performance and brain activities. As a statistic method published by Efron [32], bootstrapping approach does not depend on the normality of the sample distribution, which is a significant advantage compared with the traditional parametric statistic method. In detail, bootstrapping reestablishes the distribution of the parent samples by repeated sampling of the original samples, usually, with an iteration of 1000. In this way, the confidence interval under a certain significance level can be obtained, which can help evaluate whether the difference between two conditions is significant or not. In addition, false discovery rate (FDR) correction was performed for multiple comparisons. Further background information about the features and advantages of bootstrapping analysis is provided in Hesterberg et al. [33].

## Result

### Behavior data and participant comfort record

**Fig. 2:**
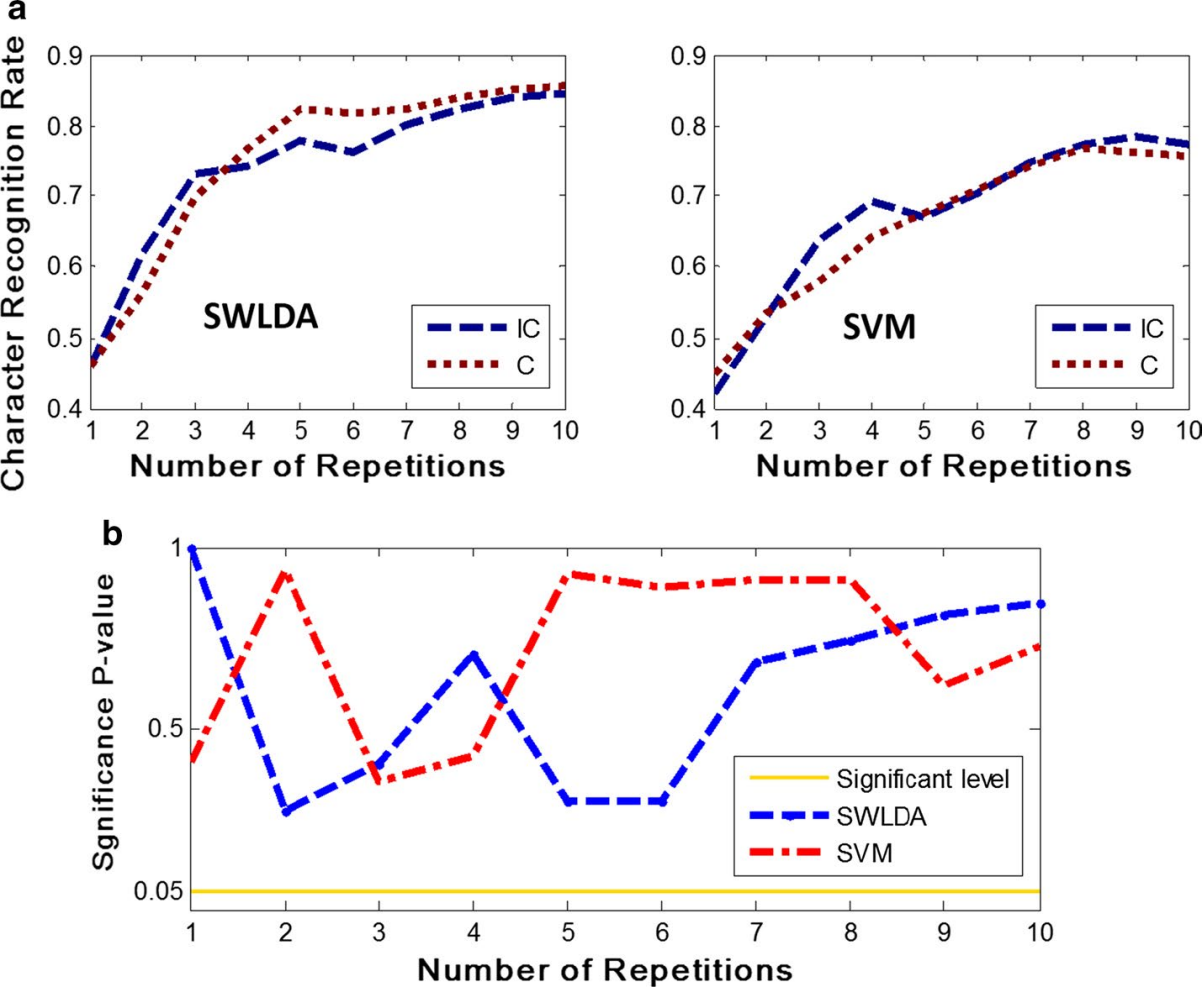
Average character recognition rate with changing number of repetitions for the IC and C paradigms. ‘C’ and ‘IC’ are the abbreviations for congruent and incongruent, respectively. The average character recognition rate obtained by SWLDA is shown on the *left* of **a**, and the average character recognition rate obtained by SVM is on the *right* of **a**. In addition, the results of the incongruent and congruent paradigm are colored *dark blue* and *dark red*, respectively. **b** Shows the significant p values of the two classification methods for the IC and C paradigms. The *blue dotted curve* represents the SWLDA results and the *red dotted curve* illustrates the SVM results. The *yellow solid curve* is the significance level of p value 0.05

Figure 2 shows the result of grand-averaged character recognition rate. Figure 2a shows the recognition rate along with the number of repetitions, and Fig. 2b shows the p values of *t* test between the recognition rates of the two paradigms. As depicted in Fig. 2a, the character recognition rate increased with the increase of the repetition number for both SWLDA and SVM classification algorithms. For further analysis, the bootstrapping *t* test was performed to compare the recognition rate between the incongruent and congruent paradigm in the two classification methods. Shown in Fig. 2b, the *t* test result suggests that the data does not reach the significance level of 0.05, and clearly demonstrates that there is no significant difference between the congruent and incongruent paradigm in the character recognition rates obtained by SWLDA and SVM.

The offline single trial classification accuracy was determined by SWLDA and SVM, and the paradigm preferences of the 11 subjects were recorded. As Table 1 shows, for incongruent stimuli paradigm, the average single trial accuracies across 11 subjects obtained by algorithms of SWLDA and SVM are 70.1 and 69.3%, respectively. For congruent stimuli paradigm, the corresponding average single trial accuracies are 67.8 and 67.6%, respectively. The result indicates little difference between the two paradigms in single trial classification accuracies obtained by both SWLDA and SVM according to Table 1, which was verified by 1000 iteration bootstrapping *t* test analysis. The single trial accuracy obtained by SWLDA of the two paradigms were compared: t(10) = 1.66, p = 0.12, and by SVM: t(10) = 1.06, p = 0.316. Overall, there is no significant difference between the paradigms in single trial classification accuracy. Additionally, the recorded comfort information indicates that most subjects preferred the semantic congruent paradigm: 8 of 11 felt more comfortable under the congruent paradigm, two found no difference between the two paradigms, and only one subject preferred the incongruent condition.

**Table 1:** Offline single trial accuracy and the paradigm preference of each subject

Subjects	Single trial accuracy (%)				Subject’s preferred paradigm		
	IC		C				
	SWLDA	SVM	SWLDA	SVM	IC	C	Either
S1	61.7	59.5	68.3	67.3		✓	
S2	67.2	65.6	62.2	60.3			✓
S3	76.6	82.7	74.5	73.8		✓	
S4	75.5	80.0	72.2	74.5		✓	
S5	63.1	61.6	58.0	57.2		✓	
S6	79.8	76.9	71.7	73.9			✓
S7	76.9	73.8	74.2	78.6		✓	
S8	77.7	78.4	73.6	73.6		✓	
S9	70.3	68.8	63.3	63.4	✓		
S10	58.9	55.0	64.4	58.1		✓	
S11	64.1	60.2	63.6	62.8		✓	
*Mean*	*70.1*	*69.3*	*67.8*	*67.6*	/	/	/

‘IC’ and ‘C’ represent incongruent and congruent, respectively. Subjects who selected ‘Either’ had no preference between the two paradigms

### ERP comparison

**Fig. 3:**
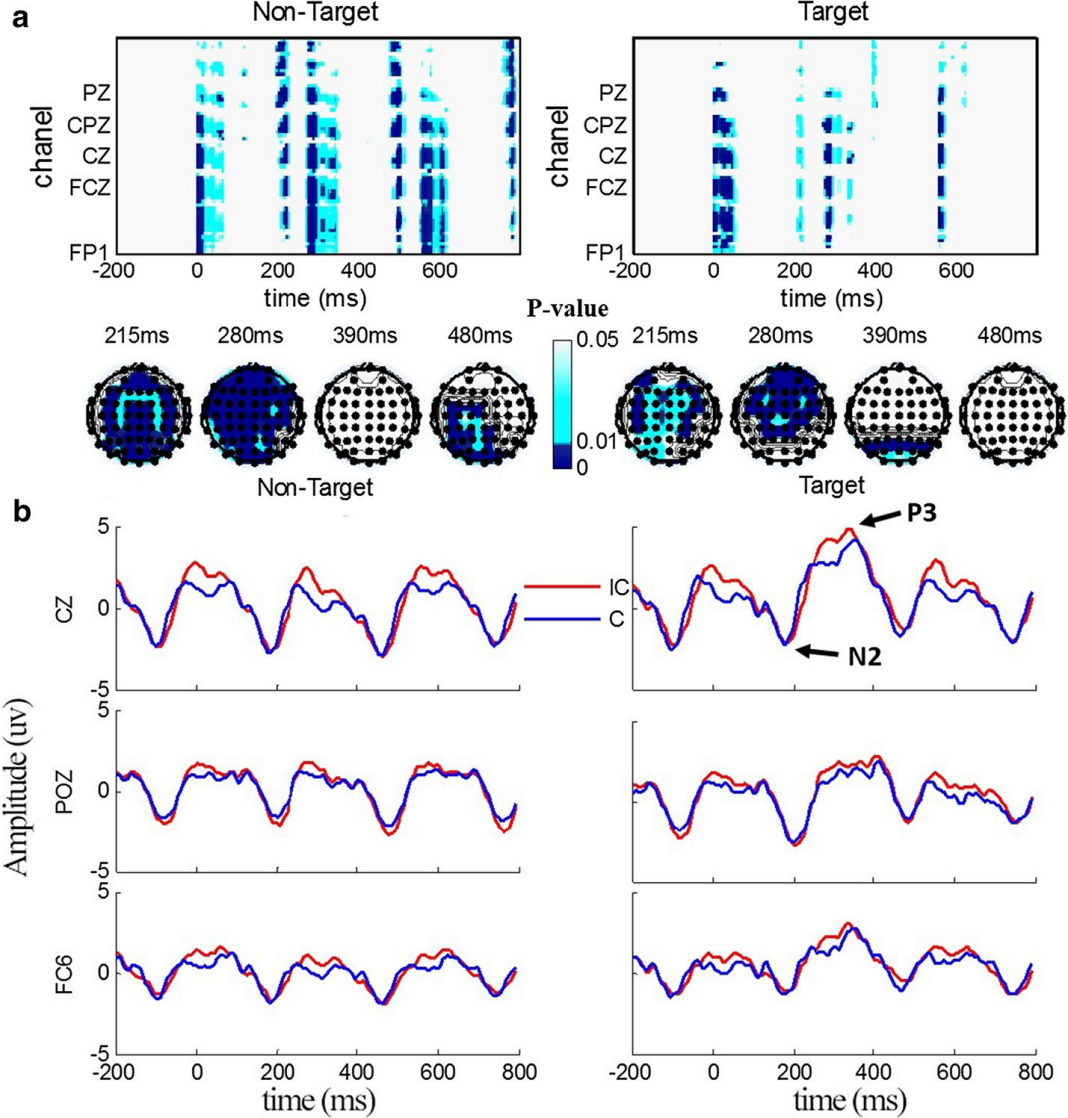
ERP comparisons between incongruent and congruent paradigm. **a** Shows the spatio-temporal distribution of p values (FDR), the *x*-axis represents time and the *y*-axis represents the channels. The comparison of non-target stimuli is on the *left* and target stimuli is on the *right* side of the figure. All plots share the same color scale shown at the *middle bottom* of **a**. *White* represents p > 0.05, *deep blue* indicates p < 0.01, and *light blue* represents 0.01 < p < 0.05. **b** Describes the grand-averaged ERP waveforms of CZ, POZ, and FC6 of 11 participants, with non-target stimuli on the *left* and target stimuli on the *right* side of **b**. The incongruent paradigm evoke dERPs are colored *red*, and the congruent paradigm evoke ERPs are colored *blue*

Figure 3a shown the significant p values over time for 62 scalp electrodes of bootstrapping comparison between ERPs of congruent and incongruent paradigm. ERPs evoked by both target and non-target stimuli were compared, and the result was corrected by FDR, since hundreds of comparisons were implemented simultaneously. To examine the differences of some ERP components in different brain regions, scalp maps of four specific time points including 200, 350, 440, and 620 ms (representing N2, P3, N4, and P5, respectively) are shown at the bottom of Fig. 3a. According to the figure, the main conclusions can be summarized as follows: from the aspect of spatio-temporal distribution difference, the paradigm type had a great influence on brain response for both target and non-target stimuli. In detail, for non-target stimuli, the significant difference was mainly observed in the posterior brain area for the time interval of 190–210 ms, the whole brain area for the time intervals of 275–300 and 490–520 ms, and the anterior brain area for the time interval of 310–340 ms. For target stimuli, there was significant difference mainly in the 200–220 ms interval for the whole brain area, 280–300 ms for the anterior brain area, and 390–410 ms for the posterior brain area. From the aspect of scalp map, the main components of ERP differed in different brain areas between target and non-target stimuli. Specifically, for non-target stimuli, there was significant difference in the whole brain area of N2, P3, and the posterior brain area of P5. For target stimuli, there was significant difference in almost the whole brain area of N2, the anterior and the center brain area of P3, and the posterior brain area of N4.

**Fig. 4:**
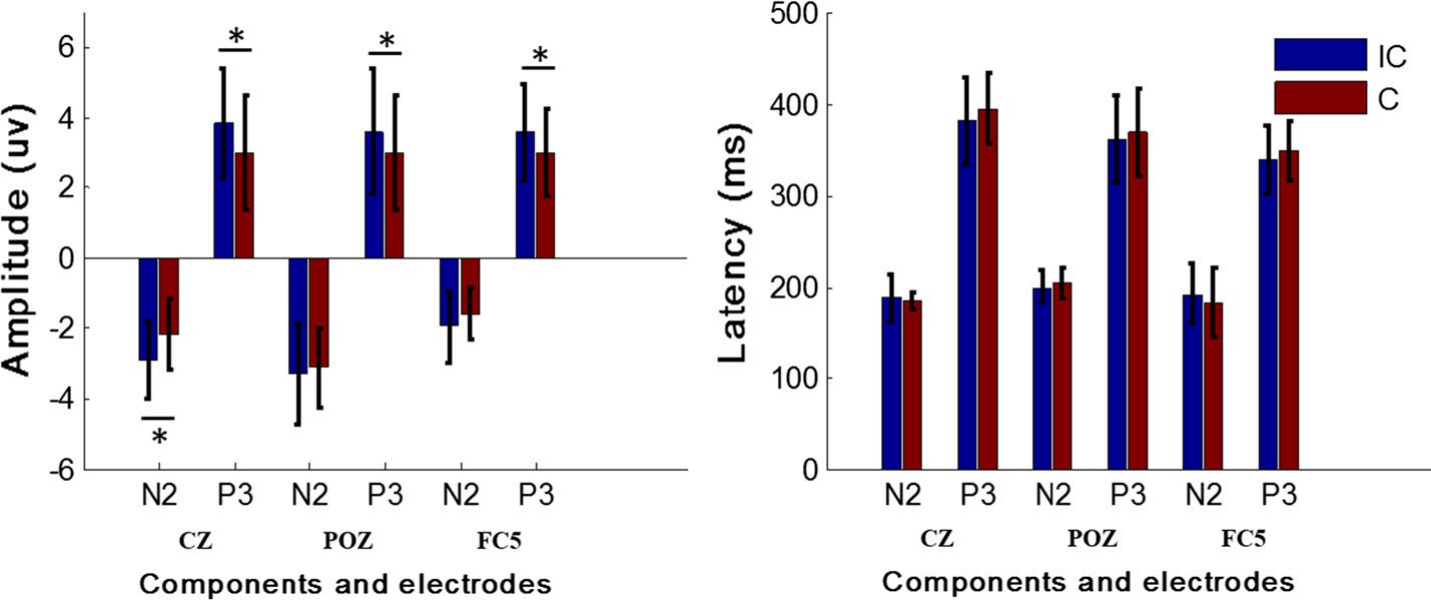
Amplitude and latency comparison of P3 and N2 for electrodes CZ, POZ, and FC5. *Asterisk* indicates p < 0.05. Results of the incongruent paradigm are colored *blue*, and the results of congruent paradigm are colored *russet*

Figure 3b depicts the grand-averaged ERP waveforms for the selected electrodes CZ, POZ, and FC6 of 11 participants for both target and non-target stimuli. As main components of ERP evoked by oddball paradigm, N2 and P3 can be observed clearly in Fig. 3b. For further analysis, the amplitude and the latency of N2 and P3 components for the three selected electrodes were selected, and 1000 iterations of the bootstrap *t* test were performed to compare the amplitude and the latency of N2 and P3 components between the two paradigms. As shown in Fig. 4, there was significant difference of amplitude at N2 of electrode CZ (t(10) = −2.56, p = 0.03), and P3 of electrodes CZ (t(10) = 2.82, p = 0.02), POZ (t(10) = 2.31, p = 0.04), and FC5 (t(10) = 3.24, p = 0.01), but no significant difference in latency was observed in both N2 and P3 for the three electrodes.

### Classifiability comparison

**Fig. 5:**
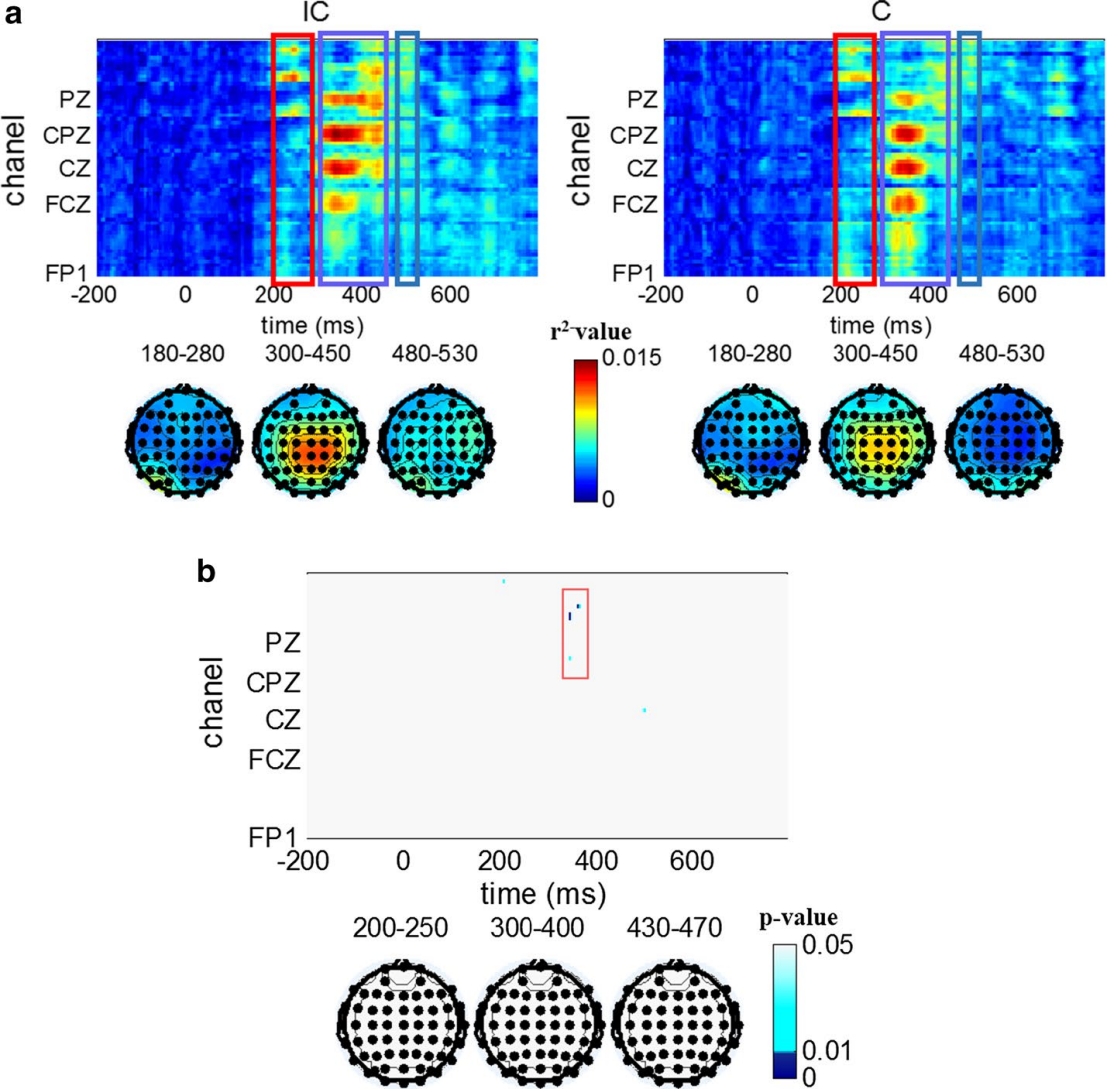
Classifiability and the comparison of classifiabilities. **a** Shows the spatio-temporal distribution of classifiabilities, where the *x*-axis represents time (ms) and the *y*-axis represents the channels. The classifiability result of the incongruent paradigm is on the *left* and the result of congruent paradigm is on the *right* side of **a**. All plots share the same color scale shown in the *middle bottom* of **a**. **b** Describes the bootstrapping (FDR) comparison of classifiability between the incongruent and congruent paradigms. The color scale is shown at the *right bottom* of **b**. *White* represents p > 0.05, *deep blue* indicates p < 0.01, and *light blue* indicates 0.01 < p < 0.05

Calculated using formula (1), the classifiabilites between target and non-target stimuli over time for all 62 electrodes are depicted in Fig. 5a. Classifiabilities were analyzed for both paradigms based on the whole dataset. The average values for three time intervals selected as features including 180–280, 300–450, and 480–530 ms are depicted as scalp maps in the bottom panel of Fig. 5a. According to the data shown in Fig. 5a, two major conclusions can be obtained: (1) from the distribution of spatio-temporal classifiability, higher classifiability values occur in the time interval of 200–500 ms for both paradigms. (2) from the aspect of scalp map, higher classifiability values were observed in the posterior brain area for 180–280 ms, and the center brain area for 300–450 ms, for both paradigms. In addition, for time interval 480–530 ms, higher values were observed in the right and left posterior brain area for the incongruent paradigm, and only the posterior brain area for the congruent paradigm.

Figure 5b depicts the bootstrapping *t* test results for classifiabilities of the incongruent and congruent paradigm. FDR correction was performed as hundreds of comparisons were implemented simultaneously. It can be clearly seen in Fig. 5b that very few significant differences are observed around PZ electrode at 370 ms approximately. Beyond that, there was little difference in time or space, and classifiability can account for the comparison result of offline classification accuracy.

## Discussion

The ERP-based speller is one of the most stable communication systems for patients with severe neuro-muscular diseases. However, there have been only few studies investigating factors that may affect system performance or user comfort for these systems. In this study, the effect of semantic congruency toward audiovisual BCI was investigated for overall system performance and participant comfort. Furthermore, high-density electrical mapping of ERPs were analyzed to explain the obtained results.

First, the *t* test result of offline classification accuracy suggested that semantic congruency between auditory and visual stimuli had little effect on system performance. However, we found an interesting phenomenon that significant larger P300 waveforms were obtained when the incongruent paradigm was applied compared with the congruent paradigm, which was completely opposite to the result that larger P300 waveforms lead to higher accuracies [34, 35]. Additionally, semantic congruency had a significant influence on participant comfort, since 8 of 11 reported that they felt more comfortable while in the semantic congruent paradigm. Semantic incongruent stimuli was a more complex paradigm in general, thus, participants must maintain increased focus and attention and stronger brain activity during an incongruent paradigm experiment compared with a congruent one. Spontaneously, a sense of discomfort emerges slowly as the experiment time increases. Furthermore, the results of ERP analysis confirmed our inference. The bootstrapping results in Fig. 3a indicated significant difference around 280–350 ms in almost the whole brain area. Additionally, the main component of ERPs, P3 and N2 were captured and compared in Fig. 4. Previous results indicated that the N2 component was associated with the interaction of auditory and visual [36]. P3 component was found to have a close relationship with workload and attention allocation for a certain task [37, 38]. Therefore, higher P3 amplitude implies stronger brain activities. These conclusions supported our experimental results directly.

As we mentioned above, there has been increased attention paid to user comfort in BCI research, resulting in this becoming an important indicator for BCI evaluations, and more generally, for human computer interface (HCI) evaluations. Kübler et al. adapted the user-centered design (UCD) concept to BCI research and development, and assessed user satisfaction with questionnaires and visual-analogue scales [39]. Ekandem et al. compared user comfort, experiment, and preparation time between two different BCI devices from an ergonomic perspective [27]. However, the quantification of user comfort remains a challenging problem because there has been no publication of a general evaluation for it. The result of our research indicated that a larger P3 amplitude of ERP corresponds to poorer participant comfort, while a smaller P3 amplitude of ERP corresponds to better participant comfort on the contrary. This phenomenon revealed a potential relationship between ERP amplitude and participant comfort, which suggested that the evaluation of user comfort in BCI might be researched from the perspective of physiological parameters.

In addition, our research found that a larger ERP amplitude did not lead to higher system performance. A large part of the reason may be that non-target stimuli elicited brain response increases simultaneously with the brain response to target stimuli, as shown in Fig. 3b. This was an interesting phenomenon and an important research issue, because it indicated that non-target stimuli can also elicit a steady brain response that differs in different stimulus paradigms. Furthermore, these findings can provide some suggestions for future design of a speller paradigm. Specifically, the design of speller paradigms should not focus solely on obtaining a large ERP amplitude. For one thing, a larger ERP amplitude may not lead to a higher system performance. For another, we might not have both better system performance and better user comfort at the same time. In another word, the design of speller paradigm must take both system performance and user comfort into consideration, and keep balance between the two factors.

Finally, it should be noted that only an offline experiment was implemented in this study, and our results lack a criterion or a detailed scale for participant comfort evaluation. To further explore the difference between the two paradigms, future studies should include an online experiment and a detailed participant comfort evaluation scale.

## Conclusion

In conclusion, this study was designed to investigate the effects of semantic congruency of auditory and visual stimuli for audiovisual speller. Behavioral data and ERP data were recorded for analysis and comparison. The result suggested that although congruency between auditory and visual stimuli in an audiovisual BCI speller had no significant effect on system performance, it had a great influence on participant comfort and the brain activities of participants. Furthermore, our study suggested that the paradigm design of spellers must take both system performance and user experience into consideration, rather than merely pursuing a larger ERP response.

## Abbreviations

BCI: brain–computer interface
ERP: event-related potential
ALS: amyotrophic lateral sclerosis
SSVEP: steady-state visual evoked potential
SMR: sensorimotor rhythms
ISI: inter stimulus interval
ICA: independent component analysis
SVM: support vector machine
SWLDA: stepwise linear discriminant analysis
IC: incongruent
C: congruent
HCI: human computer interface
UCD: user-centered design

## References

[1] Wolpaw J, Wolpaw EW (2012). Brain–computer interfaces: principles and practice.

[2] Ortega J, Asensio-Cubero J, Gan JQ, Ortiz A (2016). Classification of motor imagery tasks for BCI with multiresolution analysis and multiobjective feature selection. Biomed Eng Online.

[3] Tong J, Lin Q, Xiao R, Ding L (2016). Combining multiple features for error detection and its application in brain–computer interface. Biomed Eng Online.

[4] Mak JN, Wolpaw JR (2009). Clinical applications of brain–computer interfaces: current state and future prospects. IEEE Rev Biomed Eng.

[5] Farwell LA, Donchin E (1988). Talking off the top of your head: toward a mental prosthesis utilizing event-related brain potentials. Electroencephalogr Clin Neurophysiol.

[6] Fabiani M, Gratton G, Karis D, Donchin E (1987). Definition, identification, and reliability of measurement of the P300 component of the event-related brain potential. Adv Psychophysiol.

[7] Guo J, Gao S, Hong B (2010). An auditory brain–computer interface using active mental response. IEEE Trans Neural Syst Rehabil Eng.

[8] Baykara E, Ruf C, Fioravanti C, Käthner I, Simon N, Kleih S (2016). Effects of training and motivation on auditory P300 brain–computer interface performance. Clin Neurophysiol.

[9] Brouwer A-M, Van Erp JB (2010). A tactile P300 brain–computer interface. Front Neurosci.

[10] van der Waal M, Severens M, Geuze J, Desain P (2012). Introducing the tactile speller: an ERP-based brain–computer interface for communication. J Neural Eng.

[11] Li Y, Pan J, Wang F, Yu Z (2013). A hybrid BCI system combining P300 and SSVEP and its application to wheelchair control. IEEE Trans Biomed Eng.

[12] Li Y, Pan J, Long J, Yu T, Wang F, Yu Z (2016). Multimodal BCIs: target detection, multidimensional control, and awareness evaluation in patients with disorder of consciousness. Proc IEEE.

[13] An X, Höhne J, Ming D, Blankertz B (2014). Exploring combinations of auditory and visual stimuli for gaze-independent brain–computer interfaces. PLoS ONE.

[14] Wang F, He Y, Pan J, Xie Q, Yu R, Zhang R (2015). A novel audiovisual brain–computer interface and its application in awareness detection. Sci Rep.

[15] Chang M, Nishikawa N, Cai Z, Makino S, Rutkowski TM. Psychophysical responses comparison in spatial visual, audiovisual, and auditory BCI-spelling paradigms. In: Soft computing and intelligent systems (SCIS) and 13th international symposium on advanced intelligent systems (ISIS), 2012 joint 6th international conference on IEEE; 2012. p. 2154–7.

[16] Belitski A, Farquhar J, Desain P (2011). P300 audio-visual speller. J Neural Eng.

[17] Chang M, Nishikawa N, Struzik ZR, Mori K, Makino S, Mandic D, et al. Comparison of P300 responses in auditory, visual and audiovisual spatial speller BCI paradigms. arXiv preprint arXiv:13016360. 2013.

[18] Brunner P, Joshi S, Briskin S, Wolpaw J, Bischof H, Schalk G (2010). Does the ‘P300’speller depend on eye gaze?. J Neural Eng.

[19] Treder MS, Blankertz B (2010). (C) overt attention and visual speller design in an ERP-based brain–computer interface. Behav Brain Funct.

[20] Riccio A, Mattia D, Simione L, Olivetti M, Cincotti F (2012). Eye-gaze independent EEG-based brain–computer interfaces for communication. J Neural Eng.

[21] Andres AJ, Cardy JEO, Joanisse MF (2011). Congruency of auditory sounds and visual letters modulates mismatch negativity and P300 event-related potentials. Int J Psychophysiol.

[22] Laurienti PJ, Kraft RA, Maldjian JA, Burdette JH, Wallace MT (2004). Semantic congruence is a critical factor in multisensory behavioral performance. Exp Brain Res.

[23] Yue J, Jiang J, Zhou Z, Hu D. SMR-speller: a novel brain–computer interface spell paradigm. In: Computer research and development (ICCRD), 2011 3rd international conference on IEEE; 2011. p. 187–90.

[24] Höhne J, Krenzlin K, Dähne S, Tangermann M (2012). Natural stimuli improve auditory BCIs with respect to ergonomics and performance. J Neural Eng.

[25] An X, Ming D, Sterling D, Qi H, Blankertz B. Optimizing visual-to-auditory delay for multimodal BCI speller. In: 2014 36th Annual international conference of the IEEE engineering in medicine and biology society. IEEE; 2014. p. 1226–9. 10.1109/EMBC.2014.6943818 25570186

[26] Garcia L, Lespinet-Najib V, Saioud S, Meistermann V, Renaud S, Diaz-Pineda J, et al. Brain–computer interface: usability evaluation of different P300 speller configurations: a preliminary study. In: International work-conference on artificial neural networks. Cham: Springer; 2015. p. 98–109.

[27] Ekandem JI, Davis TA, Alvarez I, James MT, Gilbert JE (2012). Evaluating the ergonomics of BCI devices for research and experimentation. Ergonomics.

[28] Lei X, Yang P, Yao D (2009). An empirical Bayesian framework for brain–computer interfaces. IEEE Trans Neural Syst Rehabil Eng.

[29] Krusienski DJ, Sellers EW, Cabestaing F, Bayoudh S, McFarland DJ, Vaughan TM (2006). A comparison of classification techniques for the P300 speller. J Neural Eng.

[30] Chang C-C, Lin C-J (2011). LIBSVM: a library for support vector machines. ACM Trans Intell Syst Technol.

[31] Aloise F, Schettini F, Aricò P, Salinari S, Babiloni F, Cincotti F (2012). A comparison of classification techniques for a gaze-independent P300-based brain–computer interface. J Neural Eng.

[32] Efron B. Bootstrap methods: another look at the jackknife. In: Breakthroughs in statistics. New York: Springer; 1992. p. 569–93.

[33] Hesterberg T, Moore DS, Monaghan S, Clipson A, Epstein R (2005). Bootstrap methods and permutation tests. Introd Pract Stat.

[34] Lu J, Speier W, Hu X, Pouratian N (2013). The effects of stimulus timing features on P300 speller performance. Clin Neurophysiol.

[35] Gonsalvez CJ, Polich J (2002). P300 amplitude is determined by target-to-target interval. Psychophysiology.

[36] Gondan M, Röder B (2006). A new method for detecting interactions between the senses in event-related potentials. Brain Res.

[37] Kok A (1997). Event-related-potential (ERP) reflections of mental resources: a review and synthesis. Biol Psychol.

[38] Kok A (2001). On the utility of P3 amplitude as a measure of processing capacity. Psychophysiology.

[39] Kübler A, Holz EM, Riccio A, Zickler C, Kaufmann T, Kleih SC (2014). The user-centered design as novel perspective for evaluating the usability of BCI-controlled applications. PLoS ONE.

